# Proteolytic Cleavage of Receptor Tyrosine Kinases

**DOI:** 10.3390/biom11050660

**Published:** 2021-04-29

**Authors:** Hao Huang

**Affiliations:** 1 Department of Pediatric Oncology, Dana-Farber Cancer Institute, Boston, MA 02215, USA; hao_huang@dfci.harvard.edu or huanghaoacademic@gmail.com; 2 Department of Pediatrics, Harvard Medical School, Boston, MA 02115, USA

**Keywords:** receptor tyrosine kinase, proteolytic cleavage, protease, metalloprotease, secretase, caspase, cancers

## Abstract

The receptor tyrosine kinases (RTKs) are a large family of cell-surface receptors, which are essential components of signal transduction pathways. There are more than fifty human RTKs that can be grouped into multiple RTK subfamilies. RTKs mediate cellular signaling transduction, and they play important roles in the regulation of numerous cellular processes. The dysregulation of RTK signaling is related to various human diseases, including cancers. The proteolytic cleavage phenomenon has frequently been found among multiple receptor tyrosine kinases. More and more information about proteolytic cleavage in RTKs has been discovered, providing rich insight. In this review, we summarize research about different aspects of RTK cleavage, including its relation to cancer, to better elucidate this phenomenon. This review also presents proteolytic cleavage in various members of the RTKs.

## 1. Introduction

**Figure 1:**
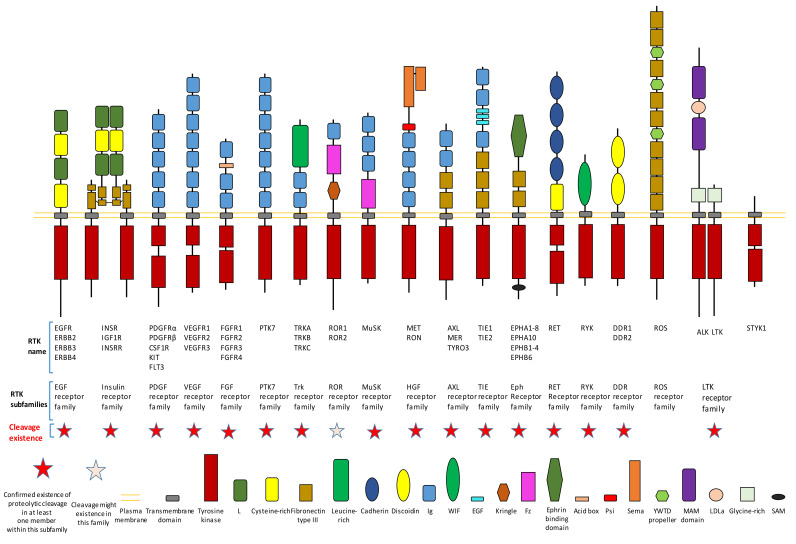
Existence of proteolytic cleavage in different subfamily of receptor tyrosine kinases.

Cell-surface receptors play a critical role in cell–cell interactions and the interactions between cells and their microenvironment. Receptor protein kinases are a large family of cell-surface receptors, which include more than 50 members (Figure 1) [1,2,3]. As cell-surface receptors, receptor tyrosine kinases (RTKs) play critical roles in signaling transmission across the plasma membrane. Therefore, RTKs play crucial roles in the regulation of many cellular processes [1,2,3]. Dysregulation of RTKs (such as aberrant activation of RTKs), which causes aberrant RTK signaling, could lead to the development and progression of diseases, such as cancers and diabetes [1,2,3,4,5,6]. Previous research has already found that aberrant activation of RTKs and their downstream signaling pathways play important roles in several cancers. RTKs’ aberrant activation could be caused by several different mechanisms, which include aberrant forms of RTKs (gain of function mutation, truncated mutation etc.), chromosomal rearrangements, genomic amplification, autocrine activation, and aberrant expression of RTK.

**Figure 2:**
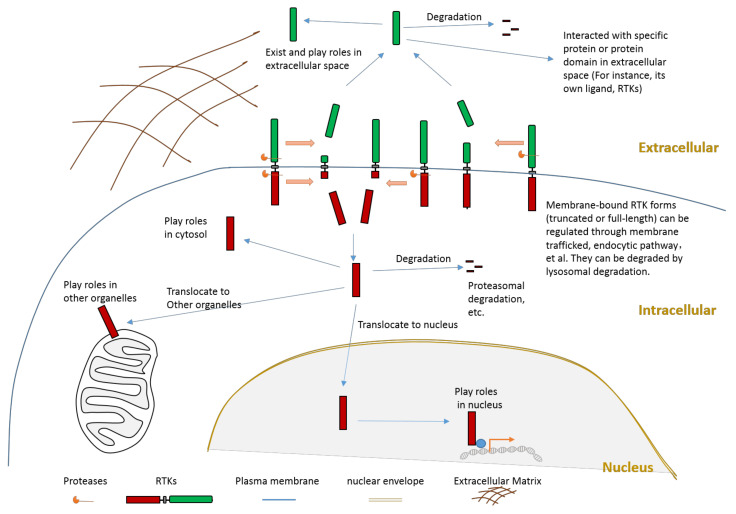
Schematic illustration about the consequences of RTK proteolytic cleavage.

It is crucial to regulate the function of RTKs since they play critical roles in signaling transduction. The regulation of RTKs function can be achieved through various mechanisms. The goal can be achieved in the following ways: mechanisms (such as transcription regulationand translation regulation) that regulate the expression of a receptor; mechanisms that affect the stability, recycling, and degradation of a receptor; the ligand-dependent activation of kinase activity; the ligand-independent activation of kinase activity; and the proteolytic cleavage of receptors. This review will focus on the introduction of proteolytic cleavage in different members of RTK. Hydrolysis of peptide bonds by proteases causes irreversible proteolytic cleavage of proteins and peptides. Proteolytic cleavage of proteins can not only affect their structure, biological function, stability, but also may influence their subcellular location and protein–protein interaction. Specific proteolytic cleavage at a particular site of protein could be viewed as a precision regulation mechanism during posttranslational modification. We must emphasize that, besides the cleavage caused by protease, other mechanisms that can also generate an intracellular truncated RTK include alternative splicing and alternative translation initiation. The objective of this review is to summarize knowledge related to RTK cleavage. This review summarizes information about the relation between RTK cleavage and cancer since cleavage may provide a alternative opportunity for targeting specific RTK in the future. This review also presents information about the cleavage of individual RTK members to better elucidate this phenomenon. After proteolytic cleavage, there are several possible consequences for those truncated fragments, as shown in Figure 2. In this review, we summarize information about the cleavage of various RTKs with the hope to improve understanding of RTKs cleavage. Reviewing the cleavage phenomena (both intracellular cleavage and extracellular cleavage) of these membrane-bound enzymes, which play critical roles in signal transduction across the plasma membrane, will provide essential and critical knowledge to a variety of fields. For example, if the relationship between the cleavage of one RTK and one specific disease is revealed, researchers may apply this knowledge to explore novel therapeutic strategies by inhibiting the cleavage of this RTK.

### 1.1. RTK Cleavage and Cancers

Proteolytic cleavage of proteins by proteases plays a critical role in numerous biological processes. The biological function of proteases is not only to catalyze the process of protein degradation to generate amino acids during protein catabolism. Another vital function of proteases is to cleave protein substrates at a specific position, which can consequently lead to some biological roles. Through proteolytic cleavage processes, proteases can regulate some important biological and physiological processes. For example, several members of RTKs, such as ALK, MET, RET, and TrkC, belong to so-called dependence receptors [7,8,9,10,11,12]. They can be cleaved by specific caspases, which then lead to the enhancement of the apoptosis process [7,8,9,10,11,12]. Therefore, dysregulation of RTK proteolytic cleavage can even cause diseases in some situations.

RTKs play critical roles in many aspects of cancer development, such as cancer cell survival, invasion, and cancer drug resistance, since they are receptors which play critical roles in signaling transduction. Tyrosine kinase inhibitors (TKI), which include many RTK inhibitors, are a large group of important cancer drugs. Several reviews have already summarized knowledge related to RTK and cancer; therefore, we will not introduce detailed information concerning this topic in this review.

For some RTK members, such as MET, specific proteolytic cleavage is a process that is closely related to cancer development [13,14]. Since RTK cleavage could result in the modification of full-length RTK production and the generation of cleaved RTK fragments which may display novel functions after translocating to a specific organelle (such as nucleus), it is not surprising to find that some specific RTK cleavage phenomena are involved in cancers. Previous research has discovered multiple biological roles of specific RTKs cleavage in cancer development. We briefly introduce the relation between RTK cleavage and cancer.

The previous result found that ERBB2 (or HER2) can be cleaved by proteases, such as ADAM10, in HER2-overexpressing breast cancer cells [15,16,17,18,19,20,21,22,23]. ECD cleavage of ERBB2 generates membrane-bound ERBB2 fragment, which can mediate the growth and survival of HER2-overexpressing breast cancer cells [15,16,17,18,19,20,21,22,23]. Moreover, the serum ERBB2 extracellular domain is emerging as a cancer marker of HER2-positive breast cancer [15,16,17,18,19,20,21,22,23].

Previous research suggests that cleavage of one ErbB4 isoform could promote cancer cell growth together with its kinase activity [24]. Cleavage of ErbB4 by γ-secretase releases a ErbB4-ICD fragment which could translocate into nucleus [25]. This fragment could interact with some transcriptional regulators in nucleus to function as a coregulator [26,27,28,29]. 

Granzyme B (GrB)-dependent intracellular cleavage of FGFR1 releases a FGFR1 fragment which can be translocated into the nucleus [30]. This cleavage could mediate breast cancer cell migration [30]. The putative mechanism is because FGFR1 fragment in the nucleus could mediates the transcription of several target genes to affect cell migration [30].

PTK7 can be sequentially cleaved by proteases (ADAM17 and γ-secretase) to generate an intracellular PTK7 fragment which can translocate into the nucleus [31]. Although the mechanism is still elusive, previous research showed that the proliferation and migration of colon cancer cells can be mediated by PTK7 cleavages [31]. Research with human fibrosarcoma HT1080 cells also suggested that cleavage of PTK7 by MT1-MMP could affect cancer cell migration and metastasis through the regulation of cell protrusions, including lamellipodia and invadopodia [32].

The RTK member MET can be cleaved by multiple proteases. Previous studies suggest that some MET cleavage is closely related to cancer [13,14,33,34,35,36,37,38,39]. Calpains could cleave MET at site around residue Arg970 to release a MET-ICD fragment (about 45 kDa) from memberane into cytoplasm [13]. This MET-ICD fragment promotes epithelial cell migration and invasion [13]. The molecular mechanism through which this MET-ICD fragment promotes cell invasion remains elusive. Previous research also suggests that soluble MET-ECD is a candidate marker of uveal melanoma [35].

AXL can be cleaved by ADAM10 and ADAM17 to release a soluble AXL-ECD into extracellular space. This soluble fragment could still bind to available AXL-ligand Gas6 through its Ig-like domains, therefore, this fragment is able to act as a decoy receptor to effectively dampen Gas6 signaling [40,41,42].

Previous research also investigated the relationship between RTKs proteolytic cleavage, TKI, and cancer drug resistance. Some studies found that, for some TKIs, differential extracellular proteolytic cleavage is a major mechanism of bypass signaling that complements other pathways of drug resistance. For example, one previous study showed that the decreased shedding of AXL (and other RTKs) in cancer cells under the stress of Mitogen-activated protein kinase inhibitor (MEKi, U0126, and PD325901) resulted in cancer drug resistance [43]. This research found that MEKi increases total and phosphorylated AXL levels on the cell surface. This study also reported that MEKi enhances the binding of TIMP1 with ADAM10, thus reducing ADAM10 activity, which results in a decrease in RTKs cleavage [43]. This study suggests that decreased RTK cleavage after treatment of MEKi causes surface RTK accumulation and enhanced mitogenic signaling through bypass kinase pathways, thus causing drug resistance [43]. In addition, previous research suggests that proteolytic cleavage of some RTKs could influence cancer cell migration [44].

Dysregulated RTK ligands shedding can also cause diseases in some situations, because this shedding may also influence RTK signaling through the release of ligands. For some RTKs, their ligands are also transmembrane proteins, which can be cleaved by specific proteases. The release of specific ligands may also involve specific proteases. For instance, a disintegrin domain and metalloproteinases (ADAMs), especially ADAM17 and ADAM10, can cleave several EGF ligands, which are ligands of EGFR [14,45,46,47,48]. Elevated EGF ligand shedding combined with EGFR amplification exists in various human cancers [49,50,51]. Additionally, deregulated RTK ligand shedding may cause the formation of deregulated feedback loops in some cases. One example of the existence of a feedback loop is from EGFR and EGF ligands [52,53,54]. Activation of the EGFR by ligands can influence the transcription of EGF ligands and ligand-shedding protease ADAM17, which, in turn, influences EGFR signaling [52,53,54]. ADAM10 is the protease that can cleave ephrins, which are the ligand of Eph receptors. Cleavage of receptor-bound ephrins can mediate receptor–ligand complexes (Eph receptor–ephrin complexes in this case) and the biological functions of these complexes [55,56,57]. ADAM12 could cleave ephrin-A1 at site Arg174-Val175 to release soluble form of ephrin-A1 from membrane [58,59]. This released ephrin-A1 could mediate lung vascular permeability and lung metastasis [58,59]. Inhibition of this cleavage or antagonization of the EphA receptors results in the decrease in lung metastasis [58,59]. We will not review research about RTK ligands’ cleavage here.

### 1.2. Some Characteristics of RTK Proteolytic Cleavage

RTK cleavage has some characteristics as a biological process that may or may not be shared by other proteolytic cleavage processes. These characteristics include: (1) this process is irreversible; (2) this process can happen in the extracellular domain, intracellular domain, juxtamembrane domain, and transmembrane domain; (3) this process can release truncated protein fragments from the plasma membrane into extracellular space or cytoplasm, which may result in the subcellular location change of these fragments; (4) sequential cleavage by various proteases can proceed with this process; (5) the biological function of an RTK as a signaling transduction receptor may be abolished by this process; and (6) in some cases, both functions of an RTK—as a receptor and as an enzyme—may be mediated by cleavage.

The proteases which conduct RTK cleavage can be grouped into several major families, including the ADAM family, matrix metalloproteinase (MMP) family, and caspase family. The ADAM family members are transmembrane multidomain proteins. ADAM family members ADAM10 and ADAM17 are frequently identified as sheddases, which conduct extracellular cleavage of RTKs. MMP family is one family of zinc-dependent endopeptidases that play critical role in the cleavage of membrane-bound proteins and extracellular matrix proteins. MMP family members are usually secreted proteases or membrane-bound proteases. MMP family members usually conduct extracellular cleavage of RTKs to release ECD fragments. Caspases are a family of cysteine proteases (endoproteases) that are involved in many biological processes, such as apoptosis, pyroptosis, and inflammation. Caspase-dependent RTK cleavage, which is closely related to apoptosis, is usually intracellular cleavage.

The proteases that conduct extracellular domain cleavage can be membrane-bound proteases (such as MT1-MMP, MT2-MMP, ADAM10, and ADAM17) or secreted proteases existing in extracellular environment (such as MMP-9 and MMP-2). The proteases that conduct intracellular cleavage can be membrane-bound proteases (such as γ-secretase) or proteases within cytoplasmic (such as caspase-3). Previous research also suggests that the transmembrane region of specific RTK members (such as CSF1R) could be cleaved by γ-secretase to release a ICD fragment [60,61,62].

## 2. Proteolytic Cleavage in Various Members of RTK

Proteolytic cleavage of RTKs can usually release some parts of its intracellular domain (RTK-ICD) or some parts of its extracellular domain (RTK-ECD) (Figure 2). After releasing from the membrane, the RTK-ECDs are shed into extracellular spaces (Figure 2). After being released from the membrane, the RTK-ICDs enter the cytoplasm. Released RTK-ICDs may be translocated into specific subcellular locations, such as the nucleus (Figure 2). Those RTK-ICDs can display their biological function as signaling molecules through distinct mechanisms. These mechanisms include, but are not limited to, interacting with transcriptional factors as co-activators, and phosphorylating target proteins. Some RTK-ICDs, such as EPHB2-ICD, can act as active signaling molecules in the cytoplasm [63]. Many RTK-ICDs, such as RYK-ICD and AXL-ICD, can translocate into the nucleus after cleavage. Moreover, for a few RTKs, the intact receptor can also be translocated to the nucleus. RTK-ICDs can also be degraded after release.

Proteolytic cleavage commonly exists in different subfamilies of RTK (Figure 1). Until now, previous research has already shown that most subfamilies of RTK contain at least one member which has the proteolytic cleavage phenomenon (Figure 1). We will introduce detailed information on proteolytic cleavage within some members of RTKs in this review.

### 2.1. AXL

**Figure 3:**
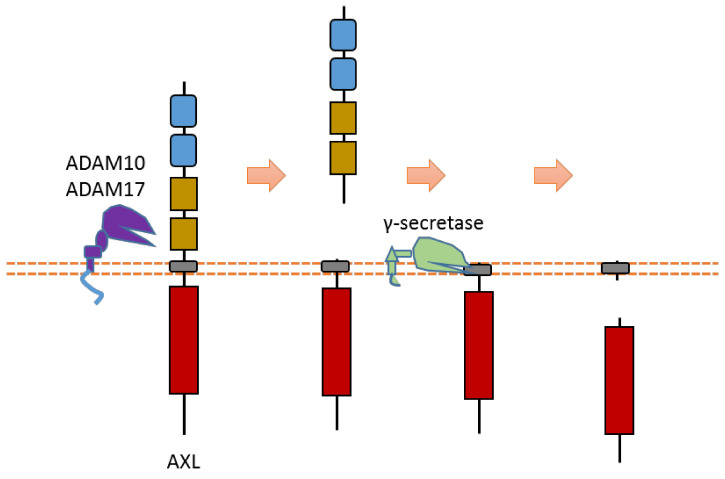
Schematic illustration of proteolytic cleavage in AXL.

AXL can be cleaved by proteases ADAM10, ADAM17, and γ-secretase [40,42,64,65] (Figure 3). Cleavage of AXL by ADAM10 and ADAM17 can release its ectodomain from the plasma membrane [40,42,64,65] (Figure 3). Cleavage of AXL by γ-secretase can release an AXL fragment from membrane-anchored AXL into the cytoplasm [64,65] (Figure 3). Previous studies suggested that the cleavage site of γ-secretase is located at the region between Tyr452 and Val459 [64,65]. The ADAM10-mediated mouse Axl cleavage site has been identified within the region between Gln426 and Ala439 (^426^QPLHHLVSEPPPRA^439^) [42]. As AXL-ICD contains a nuclear localization sequence (NLS), it could be translocated into the nucleus [64,65]. Therefore, after intracellular cleavage of AXL, AXL-ICD exists both in the cytoplasm and the nucleus.

Some research suggested that AXL cleavage may play a critical biological role [40,64,65]. For instance, one study showed that γ-secretase-uncleavable AXL mutant enhanced chemoresistance to erlotinib, an EGFR inhibitor, in non-small-cell lung cancer cells [64]. This information suggests that AXL cleavage plays a role in the cancer drug resistance of non-small-cell lung cancer cells.

MER, another member of this subfamily, can also be cleaved by ADAM17 at its ECD to release a soluble MER-ECD fragment [66]. The cleavage site was identified at Pro485-Ser486 [66].

### 2.2. MET

**Figure 4:**
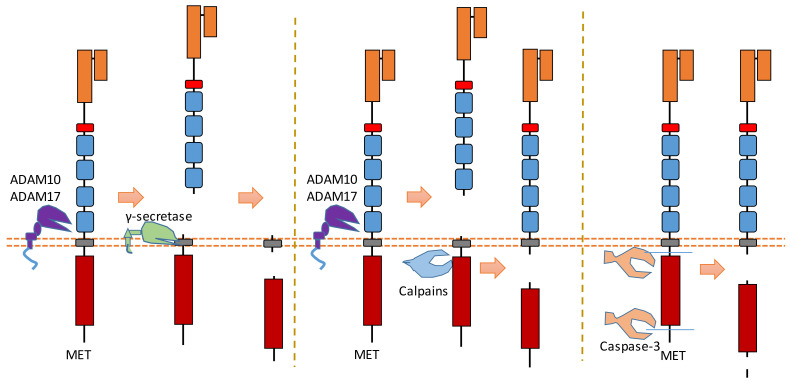
Schematic illustration of proteolytic cleavage in MET.

MET is a heterodimer composed of a 50 kDa highly glycosylated α-chain subunit and 145 kDa β-chain (Figure 4). It could be cleaved in multiple positions (Figure 4). Abnormal MET activation plays an important role in a variety of cancers.

The binding of MET to its ligand HGF causes the dimerization and activation of MET. In the MET ectodomain, ADAM 10 and ADAM 17 can cleave MET to shed the N-terminal fragment of MET [14,36,37] (Figure 4). The exact cleavage site is still not clear. These cleavages separate the Met extracellular region from its kinase domain; therefore, removing MET-ECD can inhibit MET activity and MET downstream pathways. One study suggests that ECD cleavage of MET can be inhibited by the tissue inhibitor of metalloproteinases [36].

After ECD cleavage caused by ADAMs, the generated membrane-bound MET fragment can then be cleaved by γ-secretase [38] (Figure 4). Thus, a MET-ICD (about 50 kDa) is released from the plasma membrane by the cleavage of γ-secretase. This MET-ICD can be degraded in the cytoplasm by the proteasome.

Calpains, which are calcium-dependent cysteine proteases, can also cleave MET in its intracellular domain during necrosis [39] (Figure 4). This intracellular cleavage, which also happens after ECD cleavage caused by ADAMs, releases a MET-ICD (about 40 kDa) into the cytoplasm. This cleavage site of calpain is located at Tyr1036-Ser1037 [39]. MET-ICD generated by this cleavage can also be degraded in the cytoplasm. This calpain-dependent MET cleavage at Tyr1036-Ser1037 is involved in the necrosis processes caused by MET. Additionally, calpains could also cause the intracellular cleavage of MET near the position of Arg970 [13]. This cleavage releases a MET-ICD (about 45 kDa) which can promote epithelial cell migration and invasion [13].

MET is one member of the dependence receptors [8,11]. In the absence of ligand binding, the intracellular domain of the MET receptor can be cleaved by caspase-3 to facilitate apoptosis (Figure 4) [8,11]. Caspase-dependent cleavage causes the exposure of the MET addiction/dependence domain, which is a pro-apoptotic segment [22]. Therefore, MET can enhance apoptosis after caspase-dependent cleavage within the intracellular domain. The cleavage sites of caspase-3 during apoptosis are Asp1002-Tyr1003, Asp1376-Asp1377, and Asp1380-Thr1381 [8,11]. After these two cleavages, the MET-ICD fragment is about 40 kDa. Recent research showed that this MET-ICD fragment can translocate to the mitochondria-associated membrane region [67]. This fragment triggers a calcium transfer from the endoplasmic reticulum to mitochondria, which causes mitochondrial permeabilization to promote apoptosis [67].

### 2.3. Trk A, B, C

All three members (Trk A, B, C) of the Trk subfamily have proteolytic cleavage phenomenon.

**Figure 5:**
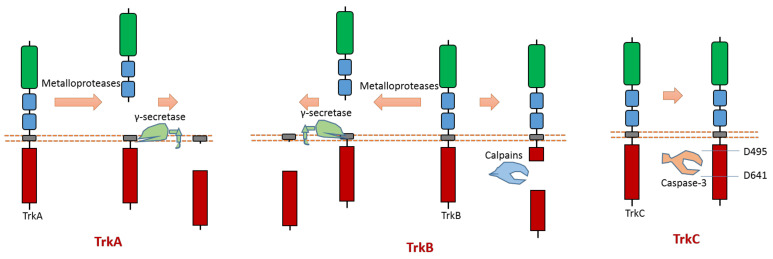
Schematic illustration of proteolytic cleavage in members of Trk subfamily.

As one member of the dependence receptor, TrkC is a substrate of caspase-3 (Figure 5). The caspase-dependent cleavage sites are located at Asp495-Asn496 and Asp641-Asn642 [9] (Figure 5). Caspase-dependent TrkC cleavage can release a pro-apoptotic domain, which can enhance apoptosis [9]. In the absence of ligand binding, TrkC can trigger apoptosis. TrkC’s pro-apoptotic activity caused by caspase-dependent TrkC cleavage may mediate sensory neuron death [9].

Recent research reported that the pro-apoptotic fragment of TrkC could interact with Cobra1, which is a cofactor of BRCA1 [68]. Cobra1 can facilitate the localization of TrkC pro-apoptotic fragment to the mitochondria to induce apoptosis [68]. TrkC pro-apoptotic fragment can induce mitochondria cytochrome c release to induce apoptosis [68]. Therefore, Cobra1 is a critical protein that is required for TrkC-induced apoptosis.

TrkA can be cleaved by metalloproteases at its ectodomain, which generates an N-terminal TrkA fragment and a membrane-anchored truncated TrkA fragment [69,70] (Figure 5). The shed fragment of TrkA is about 100 KD, and the membrane-anchored TrkA fragment is about 41 KD [69]. Research also showed that this cleavage does not occur within the endosomal–lysosomal compartments [69]. TrkA could be cleaved by γ-secretase to release a TrkA-ICD fragment [65]. The cleavage sites of proteases at TrkA are still elusive.

TrkB can be cleaved by calpain in the intracellular domain, which will generate a truncated TrkB receptor and an intracellular TrkB fragment [71,72,73] (Figure 5). It was found that amyloid-β can induce the activation of calpain, which then causes intracellular cleavage of TrkB at cleavage site Asn521-Ser522 [72]. In addition, the imbalance of full-length TrkB and the truncated TrkB amount was suggested to link to neurodegeneration [72,73]. In neurodegenerative processes, a decrease in full-length TrkB and an increase in calpain-mediated truncated TrkB were observed [73]. One specific calpain activity blocker can inhibit calpain activity; therefore, it can prevent the generation of truncated TrkB [73]. This blocker displays the ability to prevent neurodegeneration [73]. Similar to TrkA, TrkB could also be cleaved by metalloproteases at its ectodomain and γ-secretase [65,74]. Recent research found that δ-secretase, a novel secretase, could cleave TrkB at Asn365-Glu366 in the extracellular domain and at Asn486-Gly487/Asn489-Thr490 residues in the intracellular domain [75]. This δ-secretase-caused cleavage of TrkB abolishes its neurotrophic activity [75].

### 2.4. EphA2

As the largest human RTK subfamily, the Eph tyrosine kinase receptors subfamily contains more than ten members. Usually, they can regulate cell–cell and cell–ECM interaction [76]. Among them, the extracellular cleavage of EphA2 has been well studied. EphA2 is found to be expressed in several cancers [77,78,79,80]. EphA2 signaling can affect the cell–cell interaction and motility of cancer cells.

**Figure 6:**
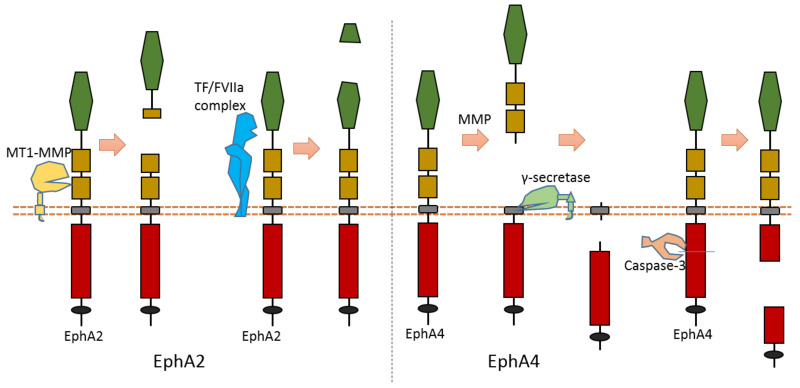
Schematic illustration of proteolytic cleavage in in EphA2 and EphA4.

Multiple proteases can cause EphA2 ectodomain cleavage (Figure 6). One study found that EphA2 can be cleaved by membrane type-1 matrix metalloproteinase (MT1-MMP) (Figure 6). The cleavage sites are at Ser431-Val432 and Ile434-Asn435, which are located at the Fibronectin type-III domain [81]. Extracellular cleavage of EphA2 by MT1-MMP can enhance cell repulsion and single-cell invasion of cancer cells [81]. Contact repulsion by membrane-associated ephrins and Eph receptors was suggested to be facilitated by ectodomain cleavage.

Another study found that the tissue factor/coagulation factor VIIa complex (TF/FVIIa complex) can cleave the extracellular domain of EphA2 and EphB2 at their ligand-binding domain [82,83] (Figure 6). Some studies suggest that this TF/FVIIa complex-induced cleavage of EphA2 can affect EphA2 signaling [82,83]. The cleavage site of EphA2 by TF/FVIIa complex is suggested to be Arg159-His160 [82].

### 2.5. EphA4

The cleavage of EphA4 has also been well studied. EphA4 could be cleaved by MMP to release an extracellular fragment [84,85]. EphA4 can be cleaved by γ-secretase to release an intracellular fragment [84,85] (Figure 6). Deletion of one juxtamembrane region (Asn533-Thr547) with 15 amino acids in EphA4 can cause resistance to cleavage, which suggests that the cleavage site is located at this region [84,85]. One research study found that ECD cleavage of EphA4 is temporally and spatially regulated during development [85]. ECD cleavage of EphA4 was found to affect cell–cell interaction and motor axon guidance [85]. EphA4 cleavage can regulate the balance of receptor–ligand interactions, which are the interactions between EphA4 and ephrin-A5. EphA4 cleavage can mediate the availability of free ephrin-As in the axon intermediate target, which is crucial to axon guidance in vivo [85]. Previous research showed that mice with blocked EphA4 cleavage have motor axon guidance defects in a mouse model [85]. Research also found that blocking EphA4 cleavage increased the expression of full-length EphA4 in limb mesenchyme. Additionally, a previous study also suggested that EphA4 can associate with ADAM10 to form a complex with ADAM10 and E-cadherin in the plasma membranes of pillar cells [86]. This study suggests that EphA4 promotes the destruction of E-cadherin-based adhesions between adjacent pillar cells [86]; and that, with the presence of Ephrin-B2, EphA4 can promote the activation of ADAM10, which results in the cleavage of E-cadherin [86]. In addition, EphA4 is a member of dependence receptor [87]. EphA4 can be cleaved by caspase-3 at Asp774-Pro775 to enhance apoptosis in the absence of ligand [87].

### 2.6. DDR1

DDR family RTKs (DDR1 and DDR2) play critical roles in cell–ECM interaction [88,89]. Some collagens can bind to DDRs as ligands to induce DDR receptor phosphorylation, which then can activate the DDR signaling transduction [88,89]. Therefore, DDRs, which are involved in the communication of cells and the extracellular microenvironment, can regulate cell–ECM adhesion, cell migration, and some other biological processes.

**Figure 7:**
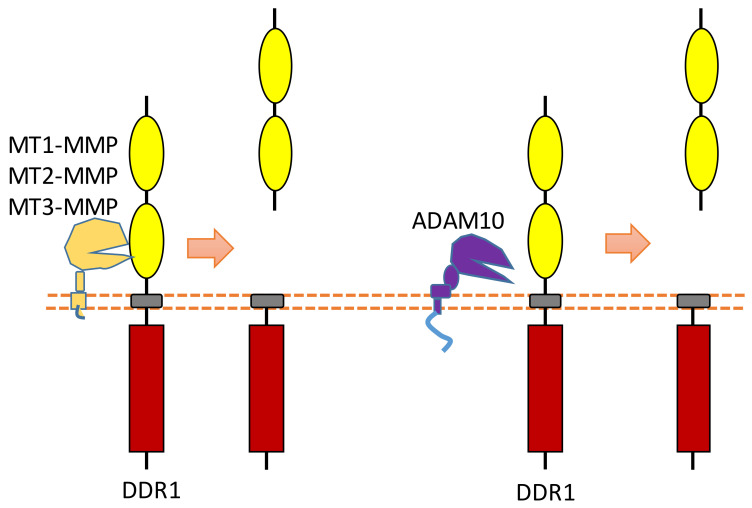
Schematic illustration of proteolytic cleavage in DDR.

Some membrane-bound protease MT-MMPs (MT1-MMP, MT2-MMP, and MT3-MMP) could cleave DDR1 in its ECD region, which can attenuate collagen I- and IV-induced receptor phosphorylation [90,91] (Figure 7). MT1-MMP can cleave DDR1 at its extracellular juxtamembrane region, which generates a membrane-bound C-terminal fragment and an N-terminal fragment [90,91]. The cleavage sites were identified at Ser397-Leu398 and Pro407-Val408 [90,91]. In addition, some research found that ADAM10 also can interact with DDR1 to cleave it when collagen is bound to DDR1 [90,92] (Figure 7).

### 2.7. FGFR1

**Figure 8:**
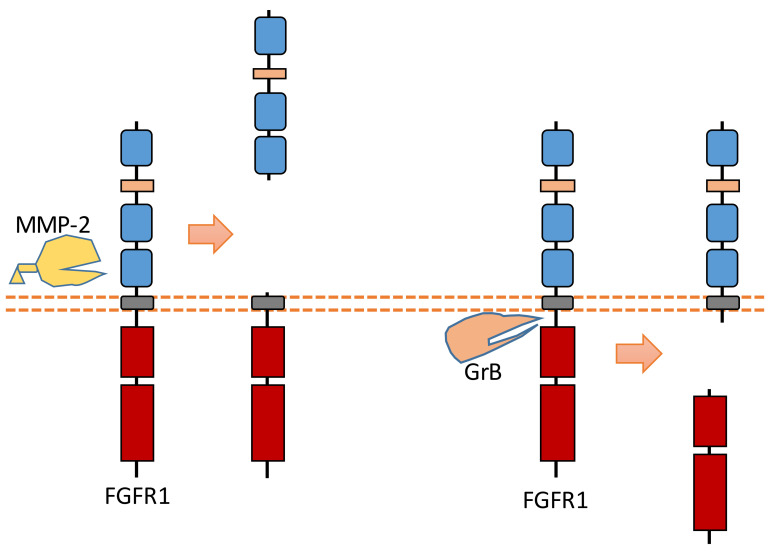
Schematic illustration of proteolytic cleavage in FGFR1.

Recent research revealed that MMP-2 could cleave FGFR1 at the nearby region of the transmembrane domain, which can shed almost the entire extracellular domain of FGFR1 [93] (Figure 8). The cleavage site of MMP-2 was identified at Val368-Met369 [93]. MMP-2-induced FGFR1 cleavage occurs no matter whether ligand FGF bind to FGFR1 or not [93]. Furthermore, the released recombinant FGFR1 ectodomain can still bind FGF after this extracellular cleavage, which indicates that this FGFR1-ECD maintained its FGF binding capacity [93].

In addition to ECD cleavage, FGFR1 can also be cleaved by granzyme B (GrB) at the intracellular domain (Figure 8). The cleavage site was identified at Asp432-Ser433 [94]. GrB-induced intracellular cleavage of FGFR1 affects the nucleus translocation of released FGFR1 fragment [30]. This cleavage can release the C-terminal fragment of FGFR1 from membrane-anchored FGFR1, and this C-terminal fragment can be translocated into the nucleus [30,94]. Research suggests that this FGFR1 fragment in nucleus could mediate the transcription of its target genes to affect breast cancer cell migration [30]. In addition to FGFR1, another FGFR subfamily member, FGFR3, can be cleaved by metalloprotease to shed its extracellular domain [95]. FGFR3 also can be cleaved by γ-secretase to release its intracellular domain [95].

### 2.8. RET

**Figure 9:**
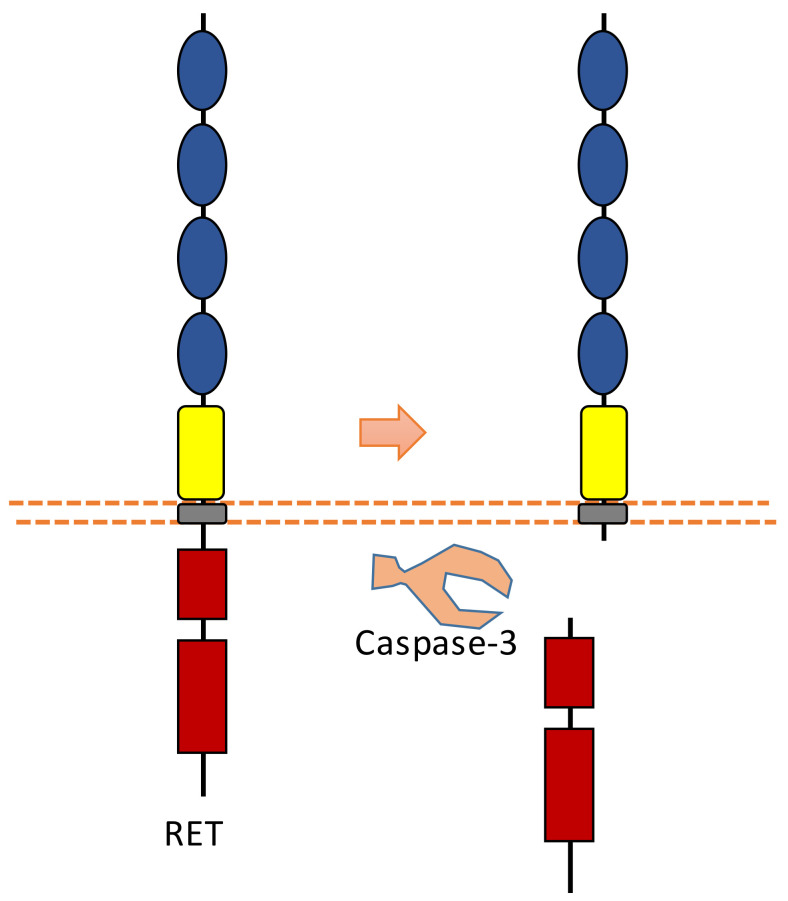
Schematic illustration of proteolytic cleavage in RET.

RET was found to be involved in the development of sympathetic neurons. RET is also a dependent receptor [7,10]. Without the binding of its ligand, caspase-dependent RET cleavage can cause apoptosis (Figure 9). The cleavage sites were suggested to be at Asp707-Ala708 and Asp1017-Leu1018 [96]. The pro-apoptotic effect of RET can be inhibited in the presence of its ligand, which is called glial cell line-derived neurotrophic factor (GDNF). With the binding of its ligand, the tyrosine kinase activity of RET is activated, which can promote survival.

Previous research also found that pro-apoptotic conditions induce RET to interact with p75, which may potentiate p75-mediated apoptosis [97]. This study showed that RET could affect nerve growth factor (NGF)-mediated survival signaling [97].

Caspase-dependent RET cleavage was found to be involved in the regulation of adhesion in sympathetic neurons [96]. This study found that after caspase-dependent intracellular RET cleavage, N-terminal truncated RET can potentiate cadherin-mediated cell aggregation [96]. As RET contains multiple cadherin domains, cleavage may increase the access of cadherin domains for cadherin-mediated adhesion.

### 2.9. ERBB4

**Figure 10:**
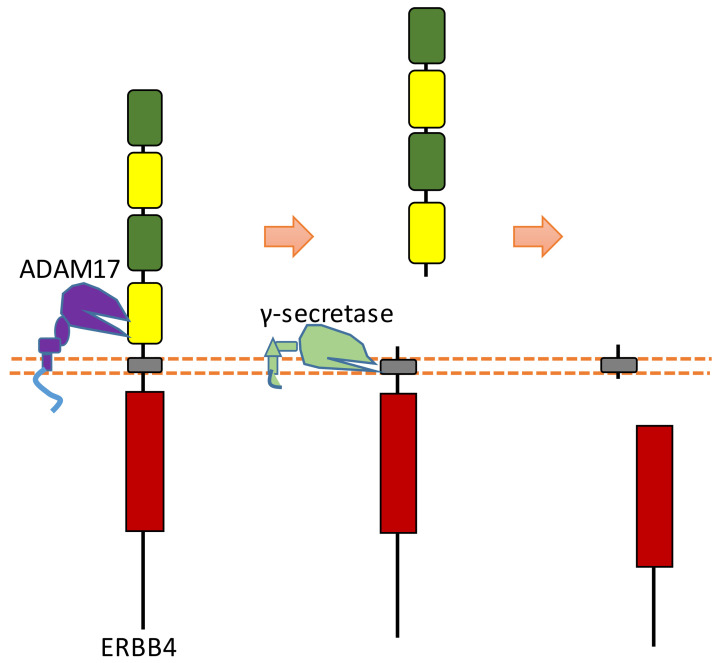
Schematic illustration of proteolytic cleavage in ERBB4.

ERBB4 can be sequentially cleaved by ADAM17 and γ-secretase in two-step cleavages, and both extracellular and intracellular fragments can be shed from ERBB4 after this two-step cleavage [25,98,99] (Figure 10). ERBB4 can be cleaved by ADAM17 at His641-Ser642 [100,101]. ERBB4-ECD can be detected in the serum. One study showed that the concentration of ERBB4-ECD in the serum of breast cancer patients is higher than that in the serum of healthy individuals [102]. ERBB4 can be cleaved by γ-secretase to release ERBB4-ICD, which can be translocated into the nucleus [25]. Previous research suggests that the cleavage sites were around the region of Val673 and Val675 [100,103]. Within the nucleus, ERBB4-ICD can interact with several transcriptional factors, such as hypoxia-inducible factor 1 alpha (HIF-1α), yes-associated protein 1 (YAP1), STAT5a, and estrogen receptor [26,27,28,29]. ERBB4-ICD can mediate transcription and gene expression because of these interactions. For instance, the interaction between ERBB4-ICD with HIF-1α in the nucleus results in the enhancement of HIF-mediated transcription, because binding with ERBB4-ICD can stabilize HIF-1α protein in both normoxic and hypoxic conditions by blocking its proteasomal degradation [29]. The biological functions of ERBB4-ICD in the nucleus are a significant consequence of ERBB4 cleavage.

### 2.10. ERBB2

As another EGF subfamily member, ERBB2, commonly referred to as HER2, can also be cleaved by proteases [15,16,17,18,19,20,21]. ECD cleavage of ERBB2 has a close relation to breast cancer [15,16,17,18,19,20,21]. Previous research indicates that ADAM10 and MMPs could cause ECD cleavage to ERBB2, which releases ERBB2-ECD fragments and creates truncated membrane-associated fragments with kinase activity [15,16,21]. Previous research suggests that the possible cleavage sites of ERBB2 were located at Arg647-Ala648, Ala644-Glu645, and Asn530-Cys531 [17,104]. Soluble truncated ERBB-ECD can be measured in the serum fraction of blood, which is related to the outcome of HER2-positive breast cancer and sensitivity to some treatments [15,16,17,18,19,20,21]. Additionally, one study suggests that matriptase could also cleave phosphorylated ERBB2 at the position around Arg558 and Arg599 [19]. This research also suggests that some membrane-associated extracellular serine proteases (such as matriptase, prostasin) could cleave the ECD of several RTKs, such as ERBB2 and ERBB4 [19]. As a member of dependence receptor, ERBB2 could be cleaved by caspases at multiple sites (Asp1016-Leu1017, Asp1019-Ala1020, Asp1087-Gly1088, and Asp1125-Gly1126) of its intrecellular domain [105].

### 2.11. ALK

ALK is one of the two members belonging to the LTK tyrosine kinase receptor subfamily [106]. ALK can be cleaved in the intracellular domain by caspase-3 [12]. Within the juxtamembrane region of ALK, there is a caspase-3 cleavage site (amino acids 1160–1163: DELD), which can be cleaved by caspase-3 to release an intracellular ALK fragment from full-length ALK [12]. Previous research showed that cleavage of ALK by caspase-3 lead to a pro-apoptosis effect because this cleavage results in the exposure of a pro-apoptotic segment (addiction/dependence domain, ADD) within the ALK juxtamembrane region [12]. Besides intracellular cleavage, ALK can also be cleaved in its extracellular domain, which generates a membrane-bound truncated ALK and a fragment of ALK released into extracellular space [107,108]. Previous studies found that ALK extracellular cleavage exists in the developing brain of rats and ALK-expressing cancer neuroblastoma.

### 2.12. Ryk

**Figure 11:**
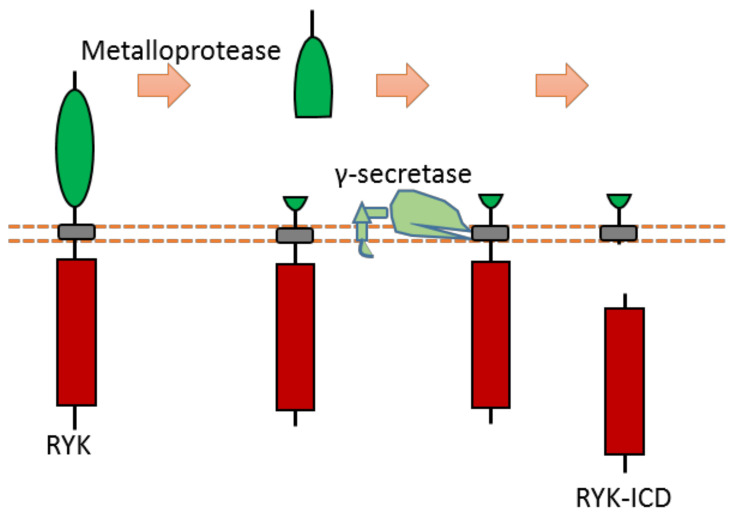
Schematic illustration of proteolytic cleavage in RYK.

As a Wnt receptor, Ryk is closely related to the nervous system, being involved in neuronal cell differentiation, axon guidance, and neurite outgrowth. Previous research suggests that the Ryk extracellular domain contains a potential proteolytic cleavage site of metalloprotease within the “KRRK” sequence motif (homo RYK Lys189-Lys192) [109,110,111]. Ryk can be cleaved by γ-secretase, which can release the ICD of Ryk from the plasma membrane [112] (Figure 11). Although the exact cleavage site of γ-secretase has not been identified yet, previous research indicates that the cleavage site is located at the transmembrane region [112]. This Ryk-ICD can be translocated into the nucleus, which was found to affect neuronal differentiation during mouse cortical neurogenesis [112]. One candidate mechanism is that Ryk-ICD may repress the neuroprotective activity of the longevity-promoting factor FOXO, which will determine neural cell fate [113]. Cdc37, a subunit of the molecular chaperone Hsp90 complex, can stabilize Ryk-ICD because it can inhibit proteasomal degradation of the Ryk-ICD [114].

Previous research shows that nuclear chaperone Smek1/2 can interact with Ryk-ICD. Smek1/2 can mediate Ryk-ICD nuclear localization [115]. This research indicates that Smek1/2 and Ryk-ICD associate with chromatin to co-occupy regulatory regions of some target genes in order to regulate their expression [115].

### 2.13. TIE1

**Figure 12:**
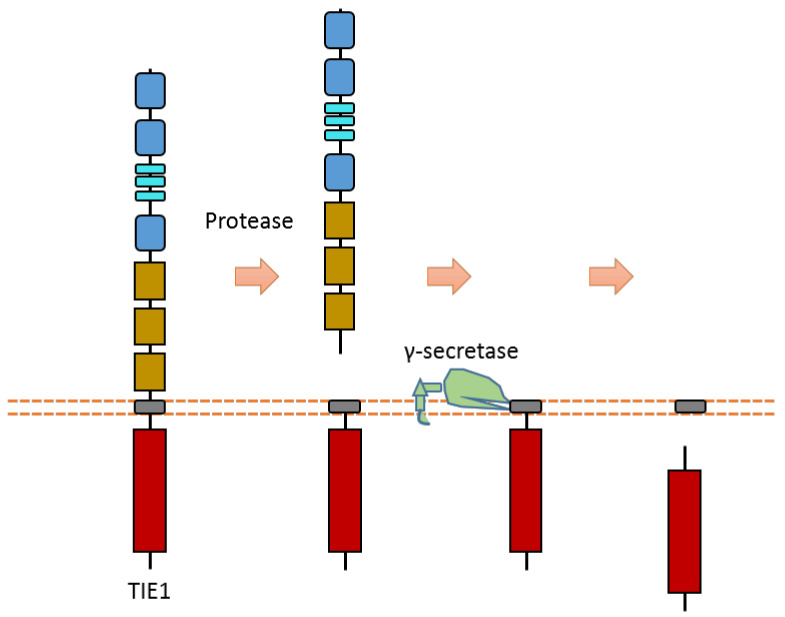
Schematic illustration of proteolytic cleavage in TIE1.

Tie1 is an RTK that is mainly expressed in endothelial cells. It involves vascular development and maintenance. Tie1 can be cleaved in the extracellular region to release the ectodomain, and then it can be cleaved by γ-secretase to release Tie1-ICD (Figure 12) [116,117,118]. The proteases and cleavage sites of Tie1 ECD cleavage are still elusive.

Tie1 and Tie2 can form the heterodimer in the plasma membrane. Some studies indicate that extracellular cleavage of Tie1 results in the modification of the ligand responsiveness of the Tie1-associated receptor Tie2 [116,117,118]. After shedding the Tie1 extracellular domain by the cleavage of Tie1, the interaction between Tie1 and Tie2 is modified [116,117,118]. This change may affect ligand–Tie2 interaction. For instance, Tie1 extracellular cleavage may cause the switch of ANG2 from a Tie2 agonist to an antagonist [116,117,118]. As Tie1 and Tie2 can interact with each other, there is also a model that indicates that the truncation of associated Tie1, after proteolytic cleavage, leads to transphosphorylation of Tie2 if it is associated with Tie1 [116,117,118]. More research is required to completely clarify the effects and mechanisms of Tie1 cleavage on angiopoietin-Tie signaling.

### 2.14. VEGFR1

VEGFR is one subfamily of RTKs that usually acts as a regulator of blood vessel function and vascular development, including angiogenesis. Some VEGFR members, such as VEGFR1 and VEGFR2, can be cleaved by specific proteases [119,120,121,122].

**Figure 13:**
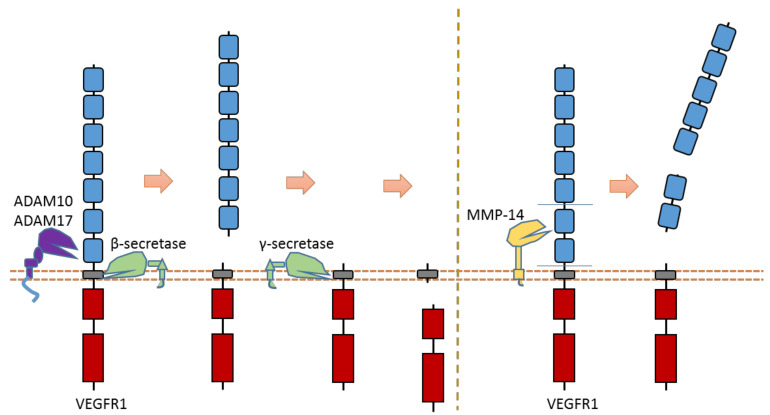
Schematic illustration of proteolytic cleavage in VEGFR1.

Recent research suggests that VEGFR1 can be cleaved by ADAMs and secretases [119,120,121] (Figure 13). ADAM10 and ADAM17 can cleave VEGFR1 to release the VEGFR1 ectodomain from the membrane [121] (Figure 13). Previous research suggests that ADAM-dependent extracellular cleavage of VEGFR1 is enhanced by protein kinase C (PKC) activation [121]. VEGFR1 can also be sequentially cleaved by β-secretase and γ-secretase [119,120] (Figure 13). Cleavage of VEGFR1 by γ-secretase can release VEGFR1-ICD into the cytoplasm of cells. γ-secretase could cleave VEGFR1 at the transmembrane region around residue Val767 [61]. The exact cleavage site of β-secretase is still elusive. Secretase-dependent VEGFR1 cleavage apprears to be able to regulate VEGF-induced angiogenesis [119,120]. Pigment epithelial-derived factor (PEDF), a known regulator of γ-secretase, can inhibit VEGF-induced angiogenesis. PEDF can enhance γ-secretase-dependent cleavage of VEGFR-1, which in turn inhibits angiogenesis [119,120]. Several studies showed that the inhibitory effect of PEDF on VEGF-induced angiogenesis can be abolished by inhibiting secretase-dependent VEGFR1 cleavage [119,120]. Additionally, previous research also indicates that MMP-14 can also cause the extracellular cleavage of VEGFR1 [123,124]. The putative MMP-14 cleavage sites were suggested at Gly558-Phe559 and around Ala744 [125].

### 2.15. PTK7

**Figure 14:**
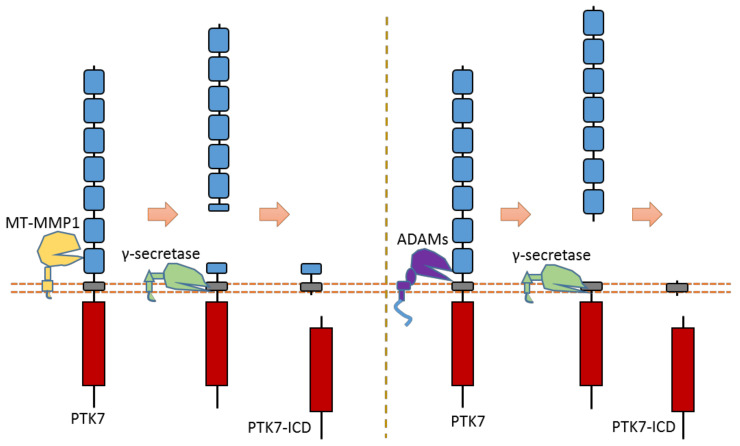
Schematic illustration of proteolytic cleavage in PTK7.

As an RTK involved in the Wnt pathway, PTK7 can regulate cell polarity and motility during embryonic development. PTK7 is an RTK that can be cleaved by at least three proteases: MT1-MMP, ADAMs, and γ-secretase [31,32,126,127] (Figure 14). MT1-MMP can cleave PTK7 at its cleavage site between P621 and L622, which is located at the seventh Ig-like domain of the PTK7 extracellular region [32,126,127] (Figure 14). ADAMs can cleave the PTK7 ectodomain at the position between Glu689 and Ser690, which is near the transmembrane domain [31] (Figure 14). After ectodomain cleavage, γ-secretase can conduct intramembrane cleavage at the position between Gly721 and Leu722 to release PTK7-CTD from the membrane [31] (Figure 14). Some studies found that PTK7-CTD, generated from cleavage, can translocate into the nucleus [31]. In addition, PTK7-CTD enhances the proliferation and migration of colon cancer cells [31].

One study found that a mutant PTK7 named chuzhoi (chz) contains an extra MT1-MMP cleavage site between the 5th and 6th Ig-like domains [126]. This mutant PTK7, which displays reduced membrane localization, can increase the cell invasion ability of HT1080 cells. This mutant was first found in mice. Mice embryos with PTK7 chz-mutant have a defective convergent extension. Another studies also found that cleavage of PTK7 by MT1-MMP could affect cancer cell motility and metastasis through the regulation of cell protrusions [32]. These studies indicate that PTK7 cleavage can mediate the PTK7 biological function related to cell motility.

### 2.16. INSR

**Figure 15:**
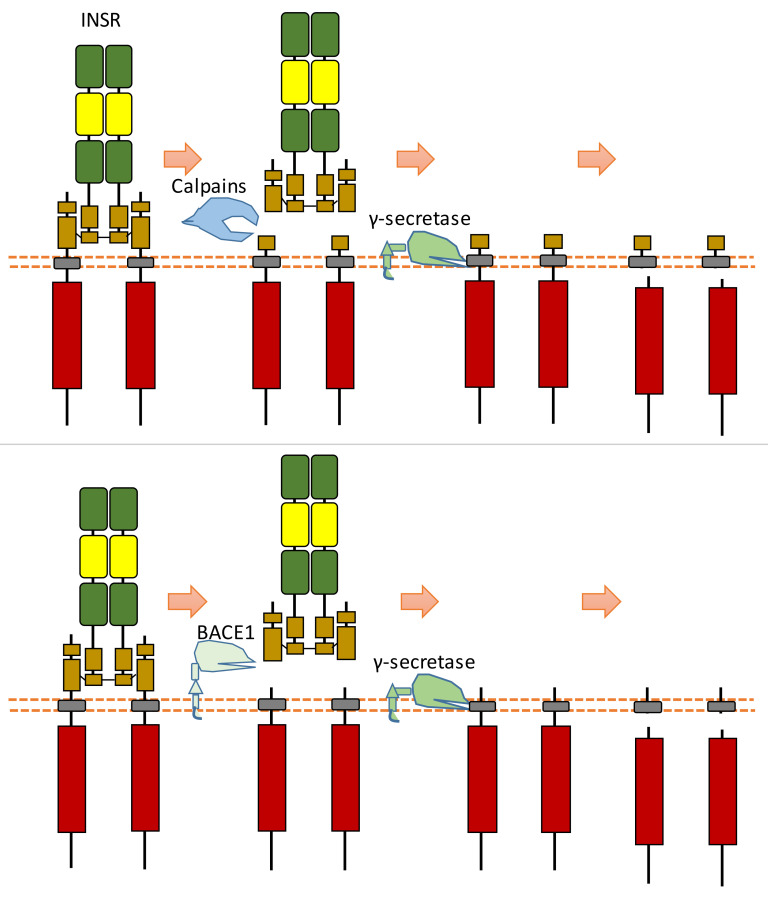
Schematic illustration of proteolytic cleavage in INSR.

In the extracellular space, calpain 2 can cleave the ectodomain of INSR (IR β-subunit), which can shed some part of the INSR ectodomain [128] (Figure 15). After this cleavage, INSR can also be cleaved by γ-secretase, which can release the C-terminal fragment of INSR [128] (Figure 15). The exact cleavage sites of proteases at INSR is still unclear. This calpain 2-dependent cleavage of INSR results in reduced insulin signaling [128]. This study found that the glucose-lowering drug metformin, which can inhibit calpain 2 release from exosomes, can lead to the inhibition of INSR cleavage and restoration of insulin signaling [128]. This study also showed that the plasma of patients with type 2 diabetes-solvable INSR levels is inversely correlated with insulin sensitivity [128].

Another research study showed that β-site amyloid precursor protein cleaving enzyme 1 (BACE1, also called beta-secretase 1) could also cleave INSR at its ectodomain [129] (Figure 15). BACE1-dependent cleavage of INSR can regulate the amount of biologically active INSR in the liver [129]. After the screening of protease inhibitors, this research found that BACE1 is involved in INSR cleavage [129]. Research on insulin receptor cleavage by BACE1 showed that BACE1 could cleave the extracellular domain of the insulin receptor [129]. This study also suggested that other proteases, such as BACE2, ADAM10, or ADAM17, do not cleave INSR in this ECD cleavage [129]. This study indicated that the BACE1-dependent INSR cleavage site is located at the membrane-proximal stalk of its β-subunits [129]. This extracellular cleavage will generate a soluble truncated form of INSR, which is composed of the α-subunits attached to part of the extracellular region of β-subunits and a transmembrane form of INSR which contains the most INSR β-subunits region. The transmembrane-truncated INSR form can be further cleaved by γ-secretase, which results in the release and degradation of ICD fragment of INSR [130,131]. Therefore, BACE1-dependent INSR cleavage can influence the amount of cell surface INSR and the amount of full-length INSR.

In previous studies, researchers found that the level of the soluble truncated form of INSR in the plasma of patients with diabetes is much higher than that of control groups [131,132]. BACE1-dependent cleavage of INSR in the liver is increased during diabetes [129,130,131,132]. The amount of biologically active full-length INSR in the liver is reduced during diabetes, which can be partially restored with the presentation of BACE1 inhibitors [129,130,131,132]. BACE1 inhibitors, which can increase the amount of full-length INSR, were also found to enhance insulin signaling [129,130,131,132]. Previous research found that glucose concentration can influence this BACE1-dependent cleavage of INSR because glucose concentration can mediate the mature of BACE [129,130,131,132]. Reducing glucose concentration decreases BACE1-dependent INSR cleavage. High glucose concentration can stimulate the level of mature BACE1, BACE1 activity, and BACE1-dependent INSR cleavage [129,130,131,132].

### 2.17. CSF1R

**Figure 16:**
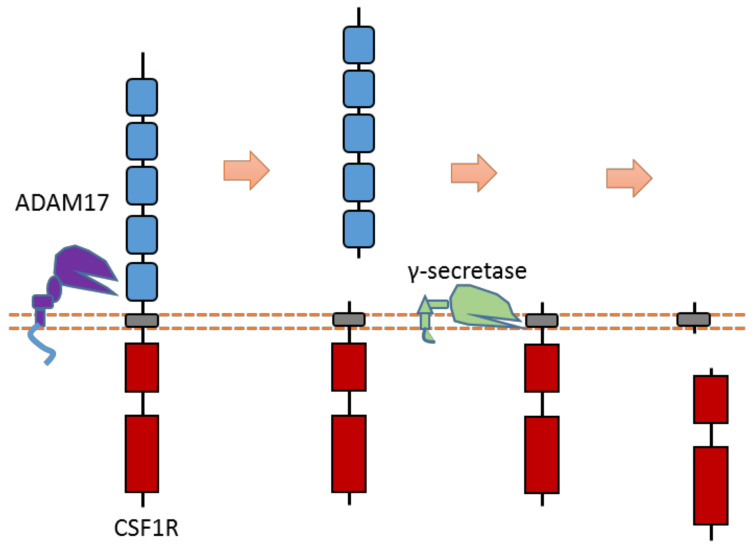
Schematic illustration of proteolytic cleavage in CSF1R.

The CSF1R receptor is one member of the PDGF subfamily RTK that could regulate the proliferation and differentiation of monocytes and macrophage precursors. CSF1R can be cleaved by ADAM17-mediated cleavage in the extracellular domain [60,62] (Figure 16). After ECD cleavage, it can then be followed by γ-secretase-mediated cleavage within the transmembrane region. The ADAM17 cleavage site of mouse CSF1R was identified at Gln503-Ser504 [60,62]. One major and one minor γ-secretase cleavage site within the transmembrane region were identified at Leu532-Leu533 and Leu535-Tyr536, respectively [60,62].

Researchers also discovered the existence of proteolytic cleavage in other PDGF subfamily members: the extracellular domain of PDGFRβ can be cleaved by ADAM10; the extracellular domain of Kit can be cleaved by ADAM17 [133,134].

## 3. Summary

Proteolytic cleavage is a common phenomenon that had already been found in many different RTK members. RTK cleavage research is an emerging research field. Systematic investigation of this biological process will help us to understand RTKs better. Most RTK cleavage is conducted by specific proteases at specific cleavage positions; therefore, regulation of RTK cleavage may be achieved through several mechanisms, including protease activity inhibition and regulation of protease activation. Knowledge about RTK cleavage may provide valuable opportunities for targeting RTK cleavage in the future.

## Data Availability

Not Applicable.
